# Anaesthetic Efficacy of 4% Articaine in Comparison with 2% Lidocaine as Intraligamentary Injections after an Ineffective Inferior Alveolar Nerve Block in Mandibular Molars with Irreversible Pulpitis: A Prospective Randomised Triple-Blind Clinical Trial

**DOI:** 10.1155/2021/6668738

**Published:** 2021-05-11

**Authors:** Nazanin Zargar, Elnaz Shooshtari, Leila Pourmusavi, Alireza Akbarzadeh Baghban, Hengameh Ashraf, Ardavan Parhizkar

**Affiliations:** ^1^Department of Endodontics, School of Dentistry, Shahid Beheshti University of Medical Sciences, Tehran, Iran; ^2^Private Practice, Kermanshah, Iran; ^3^School of Dentistry, Shahid Beheshti University of Medical Sciences, Tehran, Iran; ^4^Proteomics Research Center, Department of Biostatistics, School of Allied Medical Sciences, Shahid Beheshti University of Medical Sciences, Tehran, Iran; ^5^Iranian Centre for Endodontic Research, Research Institute for Dental Sciences, Shahid Beheshti University of Medical Sciences, Tehran, Iran

## Abstract

The objective of the current study was to compare the anaesthetic efficacy of supplemental intraligamentary (IL) injection of 4% articaine with that of 2% lidocaine in the mandibular first and second molars with irreversible pulpitis after an ineffective inferior alveolar nerve block injection (IANB) using the same anaesthetic in a randomised triple-blind clinical trial. Seventy-six adult patients, who were diagnosed with irreversible pulpitis in the mandibular first or second molars, were divided into 2 groups and received IANB randomly. In patients with lip numbness, anaesthesia was evaluated with the cold and electrical pulp (EPT) tests, and if the reported number on EPT was below 100, supplemental IL injection was administered using the same anaesthetic. The teeth were retested after 5 minutes. The Heft–Parker visual analogue scale was used to evaluate pain after IANB and IL injections. Statistical analysis was performed using repeated measures ANOVA, chi-square, and independent-sample and paired-sample *t*-tests. The results showed that there was no significant difference in the success rates of supplemental IL and IANB injections between articaine and lidocaine. Furthermore, there was no significant difference in the success rates of supplemental IL injection with lidocaine between the mandibular first and second molars. However, there was a significant difference in the success rates of supplemental IL injection with articaine between the mandibular first and second molars. Moreover, supplemental IL injections indicated no significant difference in the anaesthetic efficacy between articaine and lidocaine; nevertheless, they were more effective in the mandibular second molars, especially with articaine.

## 1. Introduction

In endodontology, pain management in intra- and postoperative stages of treatment is an important element in gaining success [1]. Lack of anaesthetic efficacy of inferior alveolar nerve block injection (IANB) for teeth diagnosed with irreversible pulpitis presents a necessity for further investigations [2, 3]. Studies have shown that maxillary teeth tend to disclose less pain during endodontic procedures when compared to mandibular teeth [4]. Moreover, researchers have reported more pain for teeth diagnosed with irreversible pulpitis/acute apical periodontitis than teeth with necrotic pulp/chronic apical periodontitis [5].

Dental professionals have been faced with the problem of obtaining proper anaesthesia while treating irreversible pulpitis [6]. IANB is the most common technique for gaining anaesthesia in mandibular posterior teeth; however, its failure is reported to be ∼10%–81%, with different reasons claimed for the lack of success [7–9]. Most recent studies have recommended several methods to overcome the anaesthetic problem, e.g., increasing the anaesthetic volume [10], decreasing the speed of injection [7, 11], using analgesics before receiving anaesthesia, and adding complements such as meperidine [12, 13]. However, application of supplemental injections, i.e., intraosseous, intraligamentary, infiltration, and intrapulpal injections, can be regarded as an effective technique/alternative to increase anaesthetic efficacy [6, 8, 14–25]. Intraosseous injection is not usually recommended due to (i) the need for special equipment and (ii) concern over increase in the heart rate when the anaesthetic contains epinephrine and levonordefrine [6, 22]. Supplemental Intraligamentary (IL) injections force anaesthetic solution(s) through the cribriform plate to marrow spaces and into the vasculature in/around the tooth, without inserting direct pressure on nerves and having periodontal ligament as their primary route [26]. A study reported the success rates of ∼37% and ∼62% for infiltration injection after an ineffective IANB in teeth with irreversible pulpitis using lidocaine and articaine, respectively [27]. In a similar investigation, a success rate of ∼63% was published for IL injection in endodontic and restorative treatments [15]. Cohen et al. reported a ∼73% success rate for supplemental IL injection in the endodontic treatment of teeth with irreversible pulpitis [28]. In other studies, the success rate for IL injection was shown to be ∼48% with lidocaine [14], ∼56% with a computer-controlled local anaesthetic delivery system [18], ∼83.33% with articaine [17], and ∼50% for primary IL injection in endodontic treatments [29]. The success rate of combining supplemental IL injection with IANB has been reported to be much higher than that of IANB alone; however, 100% success cannot be achieved [14, 15, 17, 29]. In addition, the success rate and efficacy of anaesthesia seems to be higher when the supplemental anaesthetic is the same as the anaesthetic used for IANB [8]. Furthermore, it has been reported that 4% articaine has been significantly more efficient than 2% lidocaine for supplemental buccal infiltration in the mandibular second molars with irreversible pulpitis after failed IANB [30]. Moreover, women seem to react differently to pain when compared with men and are more likely to show challenges in anaesthesia [31]. The presentation of women to avoid pain may cause anxiety, which could affect the responses to pain between women and men [32]. To the best of our knowledge, there has been no study on the comparison between lidocaine and articaine as supplemental IL injections after IANB with the same anaesthetic in mandibular molars, between the first and second molars, and between men and women.

The purpose of this study was to evaluate the anaesthetic efficacy of lidocaine supplemental IL injection after an ineffective lidocaine IANB and that of articaine supplemental IL injection after an ineffective articaine IANB in the mandibular first and second molars. In addition, the efficacy of the two anaesthetics in the mandibular first and second molars, as well as in women and men, was compared. Our null hypotheses were that there would be no difference in the anaesthetic efficacy of 4% articaine and 2% lidocaine as IL Injections after an ineffective IANB in mandibular molars with irreversible pulpitis, between mandibular first and second molars, and between women and men.

## 2. Materials and Methods

This triple-blind randomised clinical trial was approved by the Ethics Committee of Shahid Beheshti University of Medical Sciences (SBMU) (IR.SBMU.RIDS.REC.1394.63). The research protocol was registered in the Iranian Registry of Clinical Trials (IRCT) (no. IRCT2015100523253N2). The trial was registered prior to patient enrollment at IRCT (Principal investigator: Nazanin Zargar, Date of registration: 17/10/2016). Written informed consent was obtained from all patients participating in the trial.

**Figure 1:**
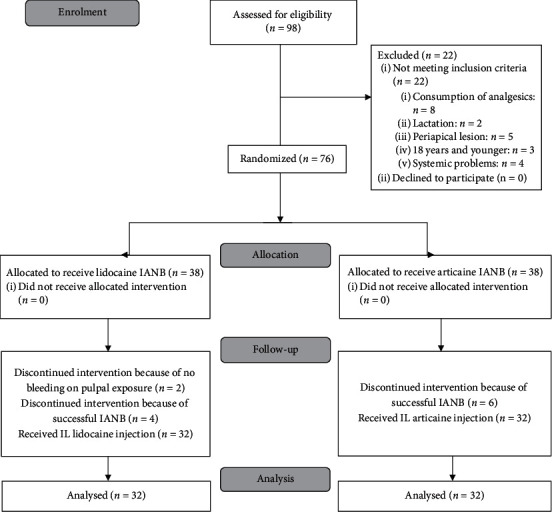
Consort flow diagram (IANB = inferior alveolar nerve block, IL = intraligamentary).

After excluding 10 individuals, owing to not meeting the initial inclusion criteria (Figure 1: Consort Flow Diagram) [33], 76 adult patients _who attended the Department of Endodontics, School of Dentistry, SBMU, Tehran, Iran, participated in the study. All patients had a vital mandibular first and/or second molar diagnosed with irreversible pulpitis. The molar teeth had no spontaneous pain; however, they showed positive response to the electric pulp test (EPT) and exhibited prolonged moderate to severe pain to the cold test with Endo ice (1,1,1,2-tetrafluoroethane; Hygenic Corp, Akron, OH). After the exclusion of 10 patients whose IANB injection was successful and 2 patients whose teeth showed no bleeding on pulpal exposure, 64 patients received supplemental IL injection including 32 men and 32 women. Inclusion criteria consisted of patients who (i) had no systemic diseases, (ii) were 18 years of age or above, (iii) had a vital mandibular first or second molar with no sign of periapical pathosis in radiographs, and (iv) did not take any pain killers 12 h prior to the treatment. Exclusion criteria composed of (a) teeth with more than 0.5 mm mobility and more than 3 mm probing depth in their mesial or distal surfaces, (b) teeth with no vital coronal pulp tissue on access, (c) patients with hypersensitivity to lidocaine or articaine, and (d) pregnancy or lactation.

To rate patients' pain, the “Heft–Parker Visual Analogue Scale” (HP-VAS) was used (i) before the beginning of the treatment, (ii) during the injections, (iii) after IANB and IL injections, (iv) throughout the preparation of access cavity, and (v) at initial filing. The HP-VAS is a 170 mm line divided into 4 categories, each showing description of a certain level of pain [34]. Pulse rates were measured using a finger pulse oximeter before and after IANB and IL injections to observe changes in the pulse rate.

Figure 1 presents the flowchart of the study events. All patients were divided into experimental groups using stratified permutations blocks randomisation. Patients were stratified into 4 groups according to the gender and type of tooth, i.e., mandibular first or second molar. Then, in every group, cases were randomly assigned to 2 subgroups of articaine and lidocaine with random block size of 4. After excluding 12 individuals (6 persons in each test group of lidocaine and articaine), 32 remaining participants of men (*n* = 16) and women (*n* = 16) received supplementary IL injection.

Equal number of articaine and lidocaine cartridges were provided and given a code. To obtain allocation concealment, only an assigned nurse was aware of the codes and randomly gave out the cartridges considering the groups (articaine or lidocaine). There was 1 code for each of the 2 cartridges packed together. One investigator completed the designated questionnaire and assessed the outcomes whereas a clinician administered/gave the injections. The clinician, who was responsible for the management of patients as well as the administration of each injection, was not involved with and/or aware of the type of anaesthetic used randomly. All the injections were administered by the same clinician. The same anaesthetic was used for block and supplemental injections. This was a triple-blind study; which means that the clinician who gave the injection, the patients, the outcome assessor, and the statistician were not aware of the used anaesthetic.

All patients received a standard IANB using a thumb ring syringe and 27-gauge long needle (Monoject; Sherwood Services, Mansfield, MA). Standard IANB was performed, starting with needle insertion [35]. After obtaining negative blood aspiration, 1.5 mL of one of the anaesthetics was slowly deposited. Then, 0.3 mL (almost one-eighth of cartridges) of the same anaesthetic was injected for long buccal anaesthesia. Half of the patients randomly received 2% lidocaine with 1 : 80,000 epinephrine (Persocaine-E; Darou Pakhsh Pharmaceutical Manufacturing Co., Tehran, Iran), and the other half received 4% articaine with 1 : 100,000 epinephrine (Septocaine; Septodont, New Castle, DE). After 15 minutes, the patients who did not report lip numbness were excluded from the study and their cartridges were replaced. The patients who reported lip numbness were selected for data analysis and entered the next step of the study.

Teeth numbness was evaluated using EPT and the cold test. EPT and cold test were both used for the confirmation of outcomes and prevention of the misinterpretation of results, i.e., false responses. Patients' pain was rated on the HP-VAS after the cold test. If the value reported by EPT was below 100, IL injection was additionally performed. The needle was inserted with the angle of 30° to the long axis of the tooth at the mesiobuccal aspect of the roots until it wedged between the tooth and crestal bone. Then, the anaesthetic (volume = 0.2 mL) was deposited into the periodontal ligament (PDL) for 20 seconds for each root. The needle remained in its position for 10 seconds after the injection. This protocol was repeated at the distobuccal aspect of the root. After 5 minutes, the teeth were examined by EPT and the cold test. Pain was recorded using the HP-VAS after the cold test.

When there was no response to EPT, the tooth was isolated and access cavity was prepared. If pain was reported during access preparation, another supplemental IL injection was given and the case was considered failure. Teeth with no pulp exposure after the removal of caries and teeth with no sign of bleeding after pulp exposure (which indicated pulp necrosis) were excluded from the study. Then, initial filing was performed. Pain during access preparation/initial filing was also recorded using the HP-VAS. When a patient reported moderate-to-severe pain during the 2 mentioned procedures, i.e., access preparation/initial filing, other anaesthetic techniques were considered and the case was considered failure. Success was defined as the ability to continue the procedures of access preparation and initial filing with no or mild pain (0 or less than 54 mm on the HP-VAS).

## 3. Statistical Analysis

The sample size calculation consisted of (i) a type 1 error of 0.05 and (ii) a type 2 error of 0.2. Calculations showed that a sample size of 64 would give 80% power to detect a 26% difference in the success rate [14, 28].

Independent- and paired-sample *t*-tests were used to compare the means of continuous variables in the designated groups. Repeated measure ANOVA followed by Bonferroni pairwise comparisons and independent- and paired-sample *t*-tests were used to compare the effects of two anaesthetics on the mean pain scores in 6-time check points. To compare the success rates between groups, genders, and mandibular first/second molars, the chi-square test was used. The significant level was set at *P* < 0.05. The analyses were carried out using SPSS 18 (SPSS Inc. Released 2009. PASW Statistics for Windows, Version 18.0. Chicago: SPSS Inc.). Furthermore, this manuscript adheres to the applicable CONSORT guideline [33].

## 4. Results

Seventy-six patients, with a mandibular first or second molar diagnosed with irreversible pulpitis, showed lip numbness after IANB injection. 10 (13.5%) IANB were successful, 6 with articaine (60%) and 4 with lidocaine (40%). However, 2 injections with lidocaine were without bleeding during access cavity preparation and were excluded. The success rates for received lidocaine IANB for the mandibular first and second molars were 30% and 7%, respectively. The success rates for received articaine IANB for the mandibular first and second molars were similar (30%).

Sixty-four patients received supplemental IL injection, 32 men and 32 women, aged between 18 and 60 years (33.33 ± 11.98). There was no significant difference between the ages (*P*=0.703) and initial levels of pain (*P*=0.989) in the two study groups. Moreover, no paresthesia was reported after articaine injection. Success rate for supplemental IL injection was 78.1% for articaine and 71.9% for lidocaine, and there was no significant difference between them (*P*=0.564) (Table 1).

The success rates of supplemental IL injection with articaine in the mandibular first and second molars were 56.3% and 100%, respectively, with a significant difference (*P*=0.007). The success rates of supplemental IL injection with lidocaine in the mandibular first and second molars were 62.5% and 81.3%, respectively, with no statistically significant difference (*P*=0.433). Therefore, as a supplemental IL injection, articaine seemed more effective in the mandibular second molars. Totally, the success rates of supplemental IL injection were 59.4% in the first and 90.6% in second molars with a significant difference (*P*=0.004). Furthermore, in the mandibular first molars, there was no significant difference between lidocaine and articaine in the mean pain scores during the preparation of access cavity (*P*=0.806) and initial filing (*P*=0.631) (Table 1).

Additionally, in the mandibular molars, there was no significant difference between articaine and lidocaine in the mean pain scores (i) during and after IANB, (ii) during and after supplemental IL injection, (iii) during access cavity preparation, and (iv) during initial filing (*P*=0.655, *P*=0.809, *P*=0.496, *P*=0.383, *P*=0.348, and *P*=0.354, respectively) (Table 2). Mandibular second molars had less pain (95% CI: 16.81 to 28.57) when compared to the first mandibular molars (95% CI: 27.56 to 44.48) (*P*=0.011). In the mandibular second molars, there was no significant difference between articaine and lidocaine in the mean pain scores during access cavity preparation (*P*=0.216) and during initial filing (*P*=0.310) (Table 2).

The success rates of supplemental IL injection with articaine for women and men were 68.80% (95% CI: 0.43 to 0.94) and 87.50% (95% CI: 0.69 to 1.06), respectively (*P*=0.394). However, they were 75.00% (95% CI: 0.51 to 0.99) and 68.80% (95% CI: 0.43 to 0.94) with lidocaine for women and men, respectively (*P*=1.00), using Fisher's Exact test. In any of the mandibular first and second molars, the check point times, and anaesthetics, there was no significant difference between men and women (*P* > 0.05).

**Figure 2:**
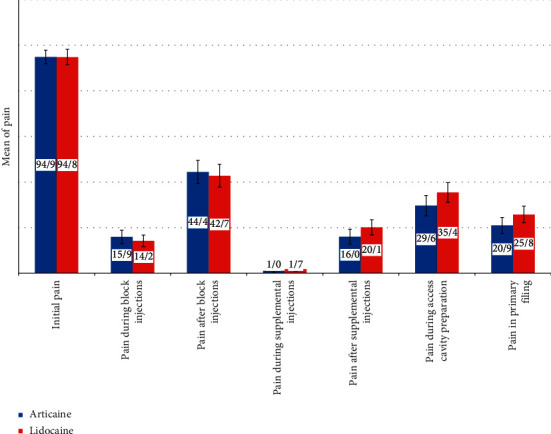
Summary of results.

There was no significant difference in the increase of pulse rate after IANB (*P*=0.628) and supplemental IL injections (*P*=0.463) between articaine and lidocaine (Table 3). However, the pulse rate increased after IANB and IL injections together. After supplemental IL injection, an increase of 3.56 and 5.03 pulses per minute was reported when using lidocaine and articaine, respectively. After IANB injection, an increase of 5.12 and 5.72 pulses per minute was reported when using lidocaine and articaine, respectively.  Figure 2 illustrates a brief summary of the obtained results.

## 5. Discussion

The present study introduces a comparison of the anaesthetic efficacy of 2% lidocaine with 1 : 80,000 epinephrine and 4% articaine with 1 : 100,000 epinephrine IL injections after an unsuccessful IANB with the same anaesthetic in the mandibular first and second molars with irreversible pulpitis; an investigation, which to the best of our knowledge, has not been conducted in the recent studies. Inferior alveolar nerve block, with supplemental injection, produces significantly higher success rate when compared to traditional IANB [20, 36]. Previous studies have shown that using supplemental anaesthetic techniques in endodontic treatments can result in successful anaesthesia after IANB failure [6, 8, 14–16, 19–25]. Amongst supplemental injections, high success rate has been reported for supplemental intraosseous injection; however, this injection is expensive, needs special equipment [21, 22], may cause tooth damage, can intensify systemic problems, and tends to produce pain/discomfort after the injection [6, 21–23, 37]. Pulpal injection requires opening of the pulp chamber and is painful [25]. Therefore, the two mentioned injections cannot be considered as first choices for supplemental injection. However, a higher success rate in endodontics has been reported when the buccal supplemental infiltration injection and IANB are performed using the same anaesthetic [8].

Our investigation showed that there was no significant difference between the success rates of IANB with the two anaesthetics as well as in the efficacy of the two anaesthetics as supplemental IL injection. However, a significant difference was observed between the mandibular first and second molars, indicating the higher success rate of IL injection in the mandibular second molars, with articaine being more effective. The aforementioned higher rate of success in the mandibular second molars could be associated with more porosity/permeability of the cribriform plate around the mandibular second molars, which may lead to the better penetration of anaesthetic(s) into the corresponding plate [26, 38, 39]. In addition, articaine has a unique thiophene ring structure causing the anaesthetic to penetrate into tissues including bone [40]. Furthermore, considering the superior lipid solubility of articaine in comparison to lidocaine, its higher efficacy is expected due to improved diffusion of the anaesthetic through nerve sheaths (e.g., inferior alveolar nerve) and/or neural membranes of individual axons [41]. Moreover, articaine shows low pKa when compared to lidocaine. The lower pKa of articaine would translate into larger percentage of the anaesthetic in the active base form. However, studies have debated low pKa as an advantage of articaine over lidocaine [42].

Moreover, there was no significant difference in the level of pain during IANB and IL injections with the two anaesthetics and between men and women. Furthermore, no significant difference was reported in the pulse rate increase between the two anaesthetics after IANB and supplemental IL injections.

The current trial revealed that there was no significant difference between the success rates of IANB with the two anaesthetics, 60% and 40% for articaine and lidocaine, respectively. Similarly, other studies have not shown a significant difference in the success rates of IANB between articaine and lidocaine [2, 3, 8, 43–45]. Also, there was no significant difference between the success rates of received articaine and lidocaine supplemental IL injections. Kaufman et al. reported 79% success rate for lidocaine IL injection using a high-pressure syringe for pulpectomy in vital teeth [46]. The higher success rate of this study may owe itself to the use of a high-pressure syringe. Walton and Abbott reported a 63% success rate for lidocaine IL injection in endodontic and restorative treatments [15]. The lower success rate of this study may be due to the difference in the type of treatment. The success rate of lidocaine supplemental IL injection using a computer-controlled local anaesthetic delivery system (CCDS) has shown to be 56% [18] in one study and 48% in another investigation [14]. The outcomes of both studies were lower than that of the present study. These differences could be due to the different distribution of the mandibular first and second molars in their study groups and between races. Another study has shown that the success rates of IL injection with CCDS in mandibular posterior teeth have been 86% for articaine and 74% for lidocaine, with no significant difference between the two study groups [47], which is similar to the results of the current study. Other investigations have revealed the success rate of IL injection with lidocaine is ∼70% [22], 74% [28], and 50–96% [24]; that conforms to the results of our study. In addition, there was no significant difference in the level of pain between lidocaine and articaine when IANB and IL injections were administered. In a study by Nusstein et al., 18% of the patients complained about moderate pain whereas only 1% of patients reported mild/no pain when the needle penetrated the buccal mucosa for the IL injection using CCDDS [18]. However, our study showed that all patients reported mild pain during IANB and IL injections; that can be due to using different injection equipment.

The present trial showed that the mandibular second molars demonstrated significantly less pain compared to the mandibular first molars, an issue that had not been investigated in the past. In IL injection, an anaesthetic is forced through the cribriform plate into the marrow space; thus, a higher success rate in the second molars could be due to the better penetration of the anaesthetic into the cribriform plate of the mandibular second molars. According to the results of this study, it seems that the cribriform plate of mandibular second molars is more permeable than that of the mandibular first molars [38, 39].

The current study used stratified randomisation (St-R) to reveal the difference between women and men. St-R expresses a situation where strata are based on the level of prognostic factors/covariates, and randomisation is applied to each stratum; for example, when there is a male participant, the subject is initially allocated to the male strata, and the group (treatment group, control group, etc.) is determined through randomisation applied to the male strata. It has been shown that stratification may increase statistical power and reduce imbalance [48]. Considering St-R, the present study showed no significant difference between men and women in the level of pain throughout all steps with the two anaesthetics. A recent study has reported a similar outcome, showing no significant difference between men and women in IL injection with articaine [49]. In addition, there was no significant difference in the increase of pulse rate after IANB and supplemental IL injections between the two anaesthetics. To the best of our knowledge, there was no study in the corresponding field.

An important limitation to the current study was the difficulty in finding/allocating desirable patients with specific needed conditions. Using a “computer-controlled local anaesthetic delivery system” seems to provide higher-volume IL injection with articaine, and thus, its efficacy and rate of success in the same/different teeth can be compared/analysed with the methodology used in the current trial.

## 6. Conclusions

The present study showed that there was no significant difference in the anaesthetic efficacy between articaine and lidocaine as supplemental IL injections in the mandibular first and second molars. However, IL injection was more effective in the mandibular second molars with irreversible pulpitis, especially the injections with articaine as the chosen anaesthetic.

## Figures and Tables

**Table 1:** Success rates and confidence intervals of supplemental intraligamentary injections with lidocaine and articaine in the mandibular first and second molars.

	Total	Lidocaine	Articaine	Success ratio ^*∗*^(95% CI)	*P* value	Effect size	Power
First molar 95% CI^1^	59.4% (19/32) 0.41 to 0.77	62.5% (10/16) 0.36 to 0.89	56.3% (9/16) 0.29 to 0.84	0.9 (0.5–1.6)	1.000	0.064	0.065
Second molar 95% CI	90.6% (29/32) 0.80 to 1.00	81.3% (13/16) 0.60 to 1.00	100% (16/16) —	1.2 (0.9–1.5)	0.226	0.322	0.445
Total 95% CI	75% (48/64) 0.64 to 0.86	71.9% (23/32) 0.63 to 0.93	78.1% (25/32) 0.55 to 0.88	1.1 (0.8–1.4)	0.564	0.072	0.089
Success ratio (95% CI)	0.6 (0.5–0.9)	0.8 (0.5–1.2)	0.6 (0.4–0.9)				
*P* value	0.004	0.433	0.007				
Effect size	0.361	0.209	0.529				
Power	0.823	0.219	0.849				

^*∗*^The success ratio of the lidocaine group to articaine group. ^1^CI: confidence interval.

**Table 2:** Summary of mean pain scores for inferior alveolar nerve block and intraligamentary injections of articaine and lidocaine in different times (mean ± SE = standard error).

Pain groups	Tooth number	Initial pain	Pain during block injections	Pain after block injections	Pain during supplemental injections	Pain after supplemental injections	Pain during access cavity preparation	Pain in primary filing
Articaine (mean ± SE)95%CI	6	92.81 ± 4.882.57–103.05	19.69 ± 4.69.86–29.51	47.81 ± 7.531.81–63.82	0.0 ± 0.0NA ^*∗*^	22.94 ± 5.311.60–34.27	40.62 ± 7.125.46–55.79	26.75 ± 6.013.82–39.68
	7	97.0 ± 3.589.42–104.58	12.13 ± 3.64.41–19.84	41.06 ± 6.826.53–55.60	2.0 ± 1.41.01–5.01	9.13 ± 2.92.76–15.49	18.63 ± 4.010.09–27.16	15.13 ± 3.18.42–21.83
	Total	94.91 ± 2.988.86–100.95	15.91 ± 2.99.87–21.95	44.4 ± 5.0234.19–54.96	1.0 ± 0.70.47–2.47	16.03 ± 3.29.41–22.65	29.63 ± 4.420.50–38.75	20.94 ± 3.513.76–28.12

Lidocaine (mean ± SE)	6	101 ± 4.990.41–111.71	14.63 ± 3.37.55–21.70	49.81 ± 7.633.56–66.06	0.63 ± 0.40.29–1.54	26.81 ± 5.115.93–37.69	45.06 ± 5.732.83–57.30	28.75 ± 5.317.42–40.08
	7	88.63 ± 4.179.79–97.46	13.69 ± 3.95.31–22.07	35.63 ± 6.222.37–48.88	2.81 ± 1.40.26–5.88	13.44 ± 3.75.43–21.44	25.81 ± 5.613.70–37.93	22.75 ± 5.111.78–33.72
	Total	94.84 ± 3.387.95–101.74	14.16 ± 2.58.99–19.32	42.72 ± 5.032.51–52.92	1.72 ± 0.70.16–3.28	20.12 ± 3.313.31–26.94	35.44 ± 4.326.60–44.27	25.75 ± 3.618.25–33.25

Total (mean ± SE)	6	96.94 ± 3.489.82–104.05	17.16 ± 2.811.38–22.93	48.81 ± 5.238.07–59.55	0.31 ± 0.20.13–0.76	24.88 ± 3.617.45–32.30	42.84 ± 4.533.64–52.05	27.75 ± 3.919.65–35.85
	7	92.81 ± 2.787.12–98.5	12.91 ± 2.67.54–18.28	38.34 ± 4.529.03–47.65	2.41 ± 0.90.38–4.44	11.28 ± 2.36.40–16.16	22.22 ± 3.415.12–29.32	18.94 ± 3.012.73–25.15
	Total	94.88 ± 2.290.42–99.33	15.03 ± 0.911.16–18.9	43.58 ± 3.536.55–50.61	1.36 ± 0.50.32–2.40	18.08 ± 2.313.43–22.72	32.53 ± 3.126.31–38.75	23.34 ± 2.518.26–28.43

^*∗*^Not applicable.

**Table 3:** Mean pulse rates before and after inferior alveolar nerve block and intraligamentary injections with articaine and lidocaine.

Variables	Technique	Mean ± SE	95% CI	*P* value
Initial pulse rate	Articaine	81.78 ± 1.503	78.72–84.85	0.860
	Lidocaine	82.16 ± 79.11	79.11–85.20	

Pulse rate after IANB	Articaine	86.81 ± 1.500	83.75–89.87	0.628
	Lidocaine	85.72 ± 1.671	82.31–89.13	

Pulse rate after IL injection	Articaine	92.53 ± 1.503	89.47–95.60	0.463
	Lidocaine	90.84–1.720	87.33–94.35	

## Data Availability

The data underlying the results, findings, and conclusions of the current study are available upon request from the corresponding author.

## Supplementary Materials

Supplementary Materials 1. Consort checklist (with page references). 2. Letter of ethics (English). 3. Letter of ethics (Persian).
