# Supplementary figures and images for: Anaesthetic Efficacy of 4% Articaine in Comparison with 2% Lidocaine as Intraligamentary Injections after an Ineffective Inferior Alveolar Nerve Block in Mandibular Molars with Irreversible Pulpitis: A Prospective Randomised Triple-Blind Clinical Trial
Source: Pain Res Manag. 2021 May 11;2021:6668738. doi: 10.1155/2021/6668738 (PMC8131152; doi:10.1155/2021/6668738)

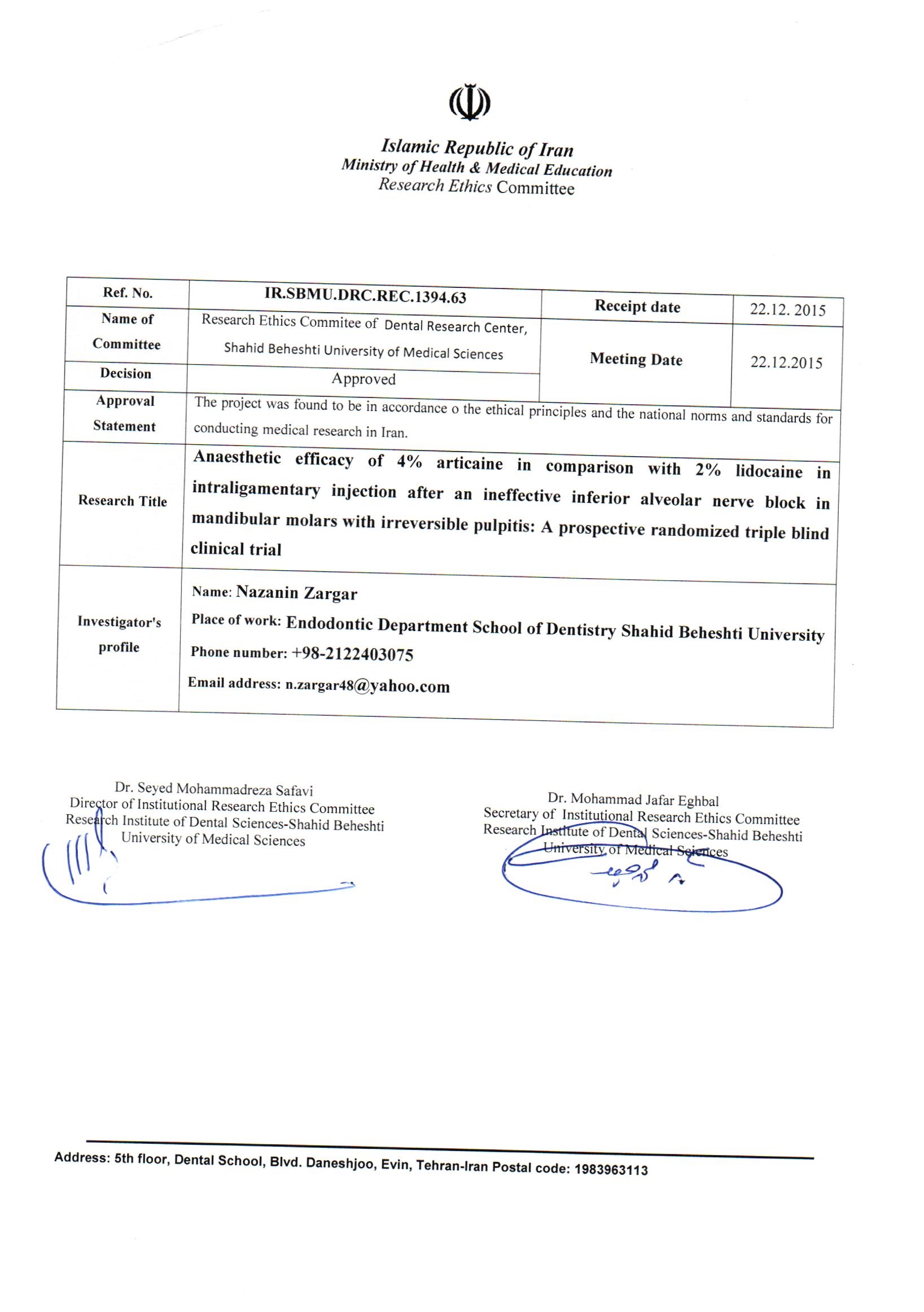

Supplement: Supplementary Materials — 1. Consort checklist (with page references). 2. Letter of ethics (English). 3. Letter of ethics (Persian). [file 6668738.f1.zip › 6668738.f1/PR&M - Letter of Ethics (English).jpg]

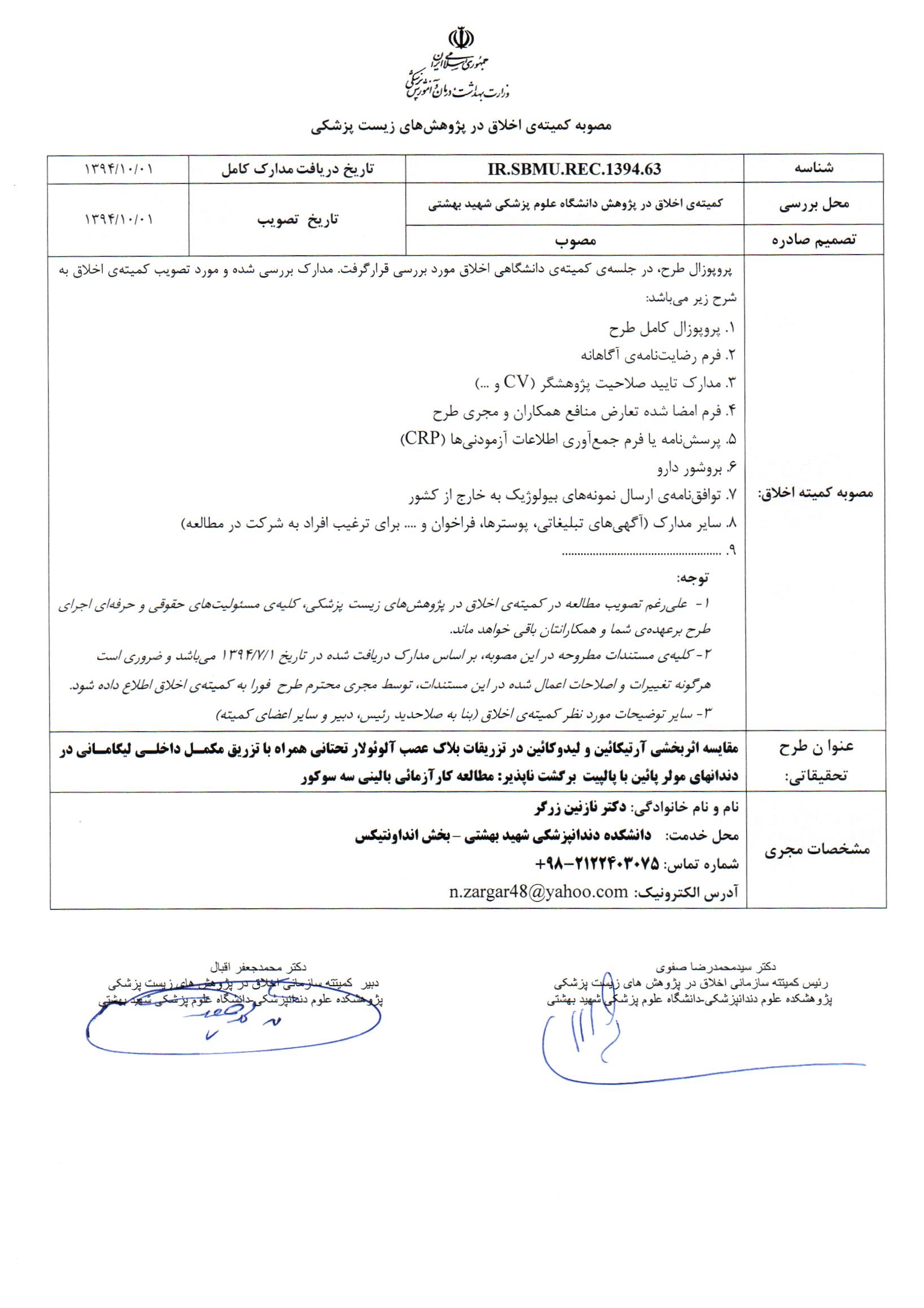

Supplement: Supplementary Materials — 1. Consort checklist (with page references). 2. Letter of ethics (English). 3. Letter of ethics (Persian). [file 6668738.f1.zip › 6668738.f1/PR&M - Letter of Ethics (Persian).jpg]
